# The Origin of Vaccine Virus

**Published:** 1848-04

**Authors:** 


*The Origin of Vaccine Virus.*—The question whether the vaccine disease in the cow is identical with the smallpox in that animal, in other words, whether smallpox communicated to the cow produces the Vaccine Virus, is still *sub-judice.* It appears to have been recently made the subject of some investigations in England to determine, if vesicles produced by inoculating the cow with smallpox virus, would yield a virus capable of producing, in the human subject, the vaccine vesicle. The results of several experiments, it is stated, establish very conclusively the identity of the Vaccine and smallpox virus, and that the former can readily be renewed by inoculating the cow with the latter. The question is one of no inconsiderable importance, as well as interest, and much credit will be justly awarded to whoever may succeed in settling it definitively. Hence, we publish with pleasure the accompanying communication, made, some weeks ago, to the Boston Medical and Surgical Journal, in which an American practitioner, Dr. Martyn, very satisfactorily establishes the priority of his experiments, and his claims to the honor of the discovery. As the experiments are interesting, we also copy in connection his former communication by Dr. Fisher, published in the Boston Journal in 1841.

The inquiry suggests itself (whether it possess any novelty or originality we cannot say) may not the vaccine disease in the cow have been primarily derived from the human subject? Its protective powers against small pox were first discovered by its having been communicated to those engaged in milking cows affected with the disease : may it not have been received by the cow, in the first instance, from the hands of the milker not fully recovered from the small pox, and afterward propagated from one animal to another by the hands of milkers? We throw out this suggestion, as it occurs to us at the moment, for what it may be worth. By Jenner, as is well known, its origin was attributed to the horse, being, as he supposed, the disease affecting horses called *grease*, communicated to the cow by inoculation.—EDITOR.

Perhaps the experiments which I made with variolous virus in October 1835, may throw some light on the subject. I resided at that time in the town of Attleborough. For this act I lost my practice, and was compelled to leave my native State and seek a new location. I removed to Greenville, Illinois, early in the spring of 1836, and remained there until the spring of 1843. I returned home in bad health, with a broken constitution. Since my return my my health has gradually improved, and I am now about to enter anew upon the duties of my profession. While at the West, I read of Dr. Creely's or Ceely's experiments, with variolous virus. I addressed a note to Dr. Fisher, of Boston, on the subject, in January, 1840. I suspected the gentleman from England had borrowed his idea from a certain guessing, experimenting *Yankee*, who had in 1835 communicated the results of his experiments to Dr. Fisher, and that Dr. Fisher had communicated the same to his friend Dr. *Creeley*, as no other individual was in the secret except Dr. Fisher; and this accounts for Dr. Creely's visiting Dr. Fisher in 1839, being four years afterwards, as appears from Fisher's letter of February 21st 1840. Ae says, " Dr. Creely, Dr. Putnam and myself, inserted various matter into the labia of the cow," &c. This may be an improvement ; it is, he is justly entitled to the credit of it, and shall have it.

I propose further to show, by facts and documents, and even the gentleman's own confession, that he is indebted to me for his idea, or, to say the least, I am entitled to priority in the discovery.

In a report made to the Rensselear Co. (N. Y.) Medical Society in 1841, Dr. Cook, (see Boston Medical and Surgical Journal, vol. xxxii., p. 49–55 and 73–75), after noticing the experiments of Dr. Sunderland and myself, says, "But probably the *most* satisfactoy, as being the most complete and best attested experiments of this kind, have been those of Mr. Robert Creely, of Aylesbury (Eng.) and his associates. Atached to a section or committee of the Provincial Medical and Surgical Association, appointed to inquire into the state of vaccination, he states that he undertook, among other experiments, to variolate the cow ; that his first trials were failures, but that early in 1839, he was more successful. He then goes on to state the manner in which he inoculated the cow, &c."

Accordingly, the committee above mentioned, engrafting the principles deduced from Mr. Creely's experiments into their joint report to the Association at Liverpool, July 25th 1839, proceed at once on the ground that the question of origin is now settled, and under the first head treat of the "affinities between," what they call "cow smallpox and human smallpox." "What many gentlemen in this country failed to accomplish, we are happy to say has been at length achieved by one of the members of our Association. Mr. Creely, influenced by some of the facts and reasons mentioned above [alluding to the experiments of Drs. Sunderland and Martin] resolved to attempt to ascertain whether he could, by inoculation, impregnate the cow with human small pox." This, to my mind, amounts to a tacit confession that he (Dr. Creely) is indebted to me for his idea of variolating the cow.

And further, the reporters designate the experiments of Mr. Creely a triumphant conclusion of an investigation of more than fifty years' duration ; and they remark, with cordial exultation, that the great problem respecting the nature of the security afforded to man by the communication of the vaccine disease is solved.

It will be perceived, from the above extracts, that Dr. Creely is to receive all the credit, and the Association at Liverpool all the honor, of settling this difficult and important question. "These things ought not to be." A humble individual of the profession has toiled hard, and suffered much, to accomplish this desirable object. And the credit and honor, if any there be, should redound to our common country, and to our medical profession in particular. It is for the profession to set this subject right, and see that there is no injustice on either side.

I will now attempt to condense the whole sum and substance, under a few general heads.

1st. I claim the discovery as original that the cow can be variolated with smallpox virus. "This is the great practical question at issue," "and this discovery puts into the hands of man an unfailing source of the vaccine virus, as long as necessity for its use shall continue.

2d. That whatever may be the nature of the virus produced, it is a safe and sure prophylactic against smallpox, when taken directly from the cow.

3d. The gentleman (from England) who has received the credit of this important discovery, is in my opinion in an error, and in consequence of building on another's foundation, had drawn false conclusions. J. C. MARTYN.

*S. Attleboro', Ms., Jan.* 8, 1848.

The following is the letter from Dr. Fisher, alluded to above.

BOSTON, Eebruary 21st, 1840.

To J. C. MARTYN, M. D.

*My Dear Sir*:—I have had the pleasure of hearing from you through your father-in-law, and also through Dr. Flagg, and be assured that I was made happy in learning that you had not fallen a lifeless victim to the shafts of enemies, or the "damning praise" of false friends; and that you are now flourishing in the great West —land of enterprise and of future greatness. It had been a long time when Dr. Flagg called on me, that I had not heard of your whereabouts, or I should have written you on the subject of our old conversation—the reproduction of the vaccine by inoculation of the cow with variolous virus.

The letter which I received from you, containing a detailed account of your experiments, I read before our Medical Club, and accompanied them with some remarks of my own upon the subject of which it treated. The reading of the paper created quite an interest among the members of the society, and you received much and deserved commendation from them for the experiments you made. Had you been a physician of Boston, your experiments would have redounded to your credit and reputation; but in the place where you now reside, it seems you only received reproaches for your laudable and scientific efforts to aid and promote the cause of science and humanity. Envy and ignorance must have been the opposition which was manifested towards you. Such feelings and and such conduct are never entertained by the medical philosopher. They can emanate only from small minds and from quacks and the supporters of quacks. They are to be despised by all high and honorable minds.

I have noticed what you say touching your experiments for the reproduction of vaccine virus, and you ought to have, and shall have, the credit of making these experiments. I believe in the original theory of Jenner—that the cowpox is the smallpox, affecting the cow.

The late experiments in England and in this city prove this to be the true theory. A Dr. Creely, of England, during the last year, has inoculated a number of cows with *variolous virus*, and produced a visicle, which contained pure *vaccine virus*—which when inserted into the arms of children, produced the true vaccine vesicle.

The same experiment has succeeded here under the hands of Dr. Putnam.

Five days ago I inoculated a cow, and a friend of mine, Dr. Whitney, of Newton, inoculated three cows, with variolous virus. What the result of these inoculations will be, I cannot yet tell. I will inform you in due season. Dr. Creely, Dr. Putnam and myself inserted the virus into the labia of the cow—just at the point where the mucous membrane begins to show itself. This is the best and most convenient place of inoculation to ensure the production of pustle. Believe me very truly your friend,

JOHN. D FISHER.

The following is the communication referred to contained in the Boston Medical and Surgical Journal, December 1, 1841.

*Experiments on the Development of Vaccine Virus.*

To the Editor of the Boston Medical and surgical Journal

SIR,—The following communication was addressed to me in a letter by John C. Martin, M. D., in 1835. Dr. Martin resided at that time in the town of Attleborough, in this State. The experiments which he made, as described in his commnnication, appeared to me to be so interesting and important, that I urged the publication of them at the time. But he declined then to publish them, on account of the excited state of feeling which his devotion to science gave rise to, in the village in which he resided. So great was the excitement of public feeling, which, I am sorry to state, was promoted in no small degree by his medical brethren of the place, that Dr. Martin lost his practice, and was compelled to seek a new location where he might practise his profession, and exert his talents for the benefit of medical science and the alleviation of human suffering. He removed to Greenville, Illinois, and has already obtained an extensive business, and established a reputation which his talents and devotion to medical science justly entitle him to. Having read of the success of the experiments instituted by Dr. Creely, of England, in re-producing the vaccine virus by inoculating cows with variolous matter, he wrote me a note in January, 1840, and requested me to hand you his statement of his experiments for publication in your Journal. I regret that his letter containing it had been mislaid, and that I have not been able, in consequence, to comply with his wish until the present time.

Dr. Martin, as well as others of the faculty, had been convinced that much of the vaccine virus in use was spurious—that having passed through so many individuals of different constitutions and habits of body, it must have lost some of its essential qualities, and that the numerous instances of the occurrence of small-pox in persons who had been vaccinated, were to be attributed to this deterioration of virus. It was this conviction that induced Dr. Martin to undertake and prosecute the experiments which he has deteiled in his paper.

He adopted the theory of Jenner, of Barron, of Sunderland, and others, "that the vaccine disease was merely the effect of the variolous poison upon the system of the cow." And had he been supported and encouraged by his professional brethren to prosecute his experiments, instead of being opposed and embarrassed, he would have gained much of the credit which has since been obtained by his distinguished professional brother in England, and would have had the honor of settling the question of the true source of the vaccine disease. The following is the communication of Dr, Martin referred to above, and should the Journal containing it be read by him, I hope he will excuse the long-delayed publication of his paper, for the reasons stated by his friend and correspondent,

BOSTON, Nov. 17, 1841. J. D. F.

*Sir*,—The following experiments may not be uninteresting to you. They were undertaken for the public good and for the benefit of science. And although I have suffered severely in mind and in purse, for making them, yet I am not sorry that the act has been committed; and all that I regret is, that I am not located in a community, and surrounded by medical men, who can duly appreciate my motives, and encourage me in prosecuting a series of experiments which I feel convinced might lead to successful and happy results.

A case of smallpox, in its worst form, having appeared in Attleborough, where I reside, and having myself, like many other physicians, failed in obtaining fresh and pure vaccine virus, and having, moreover, witnessed and read of the frequent failures of the vaccine disease, as an antidote to the attacks of smallpox, I became exceedingly desirous of obtaining the virus directly from the cow. It is true that the source of the cowpox virus is, and always has been, a matter of theory. Jenner, in his time, and many physicians of latter times, imagined, and supposed themselves to have proved, that pure vaccine matter was the result of the action of smallpox in the cow. I have been anxious to determine this point —so that should the theory prove to be true, physicians of this country might have it in their power at all times to obtain matter which would prove to be a more perfect prophylactic against variolous poison, than that which they are now obliged to use.

In order to test the theory fairly, I purchased a fat, healthy cow, eight years old, shut her in a stable, and fed her scantily for a few days. I then obtained some variolous matter from the individual who was sick with the smallpox, and who had been laboring under the disease eleven days. With this matter I inoculated the cow on the 2d day of October, 1835, in the following manner. I made, with a common lancet, fourteen or fifteen pnnctures in one of her teats between the cuticle and true skin, taking care not to draw blood. I then inserted into these various punctures, quills charged with the variolous virus. The wounds soon disappeared, and presented no appearance of being variolated until five days. On the *fifth* day the animal seemed to show some indisposition, and on examining her teat I discovered one small elevated spot at the point of insertion of one of the quills, and an evident febrile heat in the teat, when compared with those not inoculated. This increased febrile heat continued for about forty-eight hours, and then subsided. During this time the animal lost her appetite, became thirsty, her milk ceased to be secreted, and her teat began to swell.

On the *eighth* and *ninth* days a regular pustule was formed at the point inoculated, the margin of which contained some thin, light straw-colored fluid. On the *tenth day* the pustule had increased in size and become more prominent, and was distended with matter. At this period it was not regularly round, but presented an uneven surface. On the *eleventh day*, and evident change had taken place in the appearance of the pustule, it having begun suddenly to dry up. On the *thirteenth day* the virus had become solid, so that the pustule was converted into a crust, or scab, of a dark-brown color.

Besides introducing the smallpox virus into the udder, I inserted some also into a puncture which I made on the hip of the animal. This resulted in a sore and in the falling off of the hair. This inoculation produced no pustule or eruption, save at the point of insertion, so far as I could discover.

I now determined to insert some of this new generated matter into the human system, and observe its effects. Accordingly, I selected a healthy boy, aged 10 years, for the subject of my first experiment; and on the evening of the 12th day of October (the day I took some virus from the cow, being the 10th day of the existence of the pustule), I inserted some of the virus into the boy's arm in the same manner as in practising common vaccination. The symptoms resulting from the operation were the following. The virus lay dormant four days. On the *fifth day* a slight inflammation or red spot arose around the point of insertion. From this period the vesicle ran its course, like the common vaccine vesicle, was characterized by a well-formed and regular areola, and in due time was transformed into a perfectly round, mahogany-colored scab. The boy exhibited but little indisposition during the course of the disease, except headache, and he continued to play with his fellows about the street, and I saw no symptoms in his case which do not attend the vaccine dissease in its various stages. It should be mentioned, however, that two or three small pimples appeared on the boy's face and arm. These did not fill, but soon dried and disappeared.

While observing the rise and progress of this disease, I had no doubt that the eruption was like, and that it was, the true and perfect vaccine vesicle. In order that I might not be deceived, however, I took the boy to Providence, and exhibited his arm to two physicians of that place, Drs. Brownell and Toby, both of whom pronounced the eruption to be the perfect vaccine, and gave me their opinion in writing to this effect. Having satisfied myself of the nature of the eruption produced in the boy's arm, I took matter from it on the *seventh day*, and inserted some of it in the arm of my own child, which was five months old. On the *fourth day* a red spot appeared around the point of insertion, a vesicle was formed and observed the same course, and presented the same appearances, as did that on the boy's arm, from which the virus was taken. The areola, perhaps, was not quite so regular as in the case of the boy—and the febrile excitement was greater in the child, which I attributed to its natural irritability of temperament. There were in this case a number of secondary eruptions on the surface, resulting from the vaccination.

The *third subject* vaccinnated was also an infant. The child I vaccinnated with matter from my child's arm on the seventh day of the disease. This child's symptoms were similar to those presented by my own child; they were perhaps rather more severe. The areola was not so perfect, and there appeared on it a greater number of secondary vesicles which became filled with fluid. The *fourth* individual vaccinated, was a boy four years old. The virus with which I vaccinated him was taken from the arm of the child whose case I last described. The symptoms attending this case were similar to those presented in the preceding one—except that they were more severe. The areola, however, was not so regular, and the vesicle was rather more imperfect, and a greater number of secondary eruptions presented themselves on the body—some of which filled and formed crusts.

I will not trouble you further in describing cases. The whole number of persons I vaccinated was twenty-three, and the cases above described will give you a notion of the character and progress of the others. I will remark, however, that I think the last individual vaccinated had the disease more severely, as the matter used in producing it was more remotely related to the cow.

Such have been the results of my experiments, and I should feel highly gratified and honored with your opinion respecting them.

Very truly your friend, J. C. Martin.

